# Isolated cutaneous metastasis of uterine leiomyosarcoma: *case report and review of literature*

**DOI:** 10.1186/1746-1596-7-85

**Published:** 2012-07-18

**Authors:** Shane Corcoran, Aisling M Hogan, Tamas Nemeth, Fadel Bennani, Francis J Sullivan, Waqar Khan, Kevin Barry

**Affiliations:** 1 Department of Surgery, Mayo General Hospital, Castlebar, Co. Mayo, Ireland; 2 Department of Histopathology, Mayo General Hospital, Castlebar, Co. Mayo, Ireland; 3 Department of Radiation Oncology, Galway University Hospital, Galway, Ireland

**Keywords:** Sarcoma, Leiomyosarcoma, Uterine leiomyosarcoma, ULMS, Metastasis, Metastatic uterine leiomyosarcoma, Scalp, Cutaneous metastasis, Atypical metastasis, Progesterone receptor, PR, p53

## Abstract

**Abstract:**

A 54 year old lady presented for routine excision of a scalp lesion thought clinically to represent a sebaceous cyst of the right occiput. 4 years earlier she underwent total abdominal hysterectomy and right salpingo-oophorectomy for 3 large uterine fibroids. Histo-pathological examination of the hysterectomy specimen revealed an incidental low-grade leiomyosarcoma. Staging imaging was negative for metastatic disease. She made an uneventful recovery and was treated further by adjuvant pelvic radiotherapy.

She noticed an uncomfortable and unsightly cystic swelling on her occiput four years after hysterectomy and was referred for routine excision of what was believed to be a benign lesion. The lesion was excised and sent for histopathological examination. Microscopic analysis including immuno-histochemistry demonstrated an ER and PR positive metastatic deposit of leiomyosarcoma. The margins of excision were histologically clear of disease.

At Multi-Disciplinary Team (MDT) discussion a diagnosis of metastatic scalp deposit from previous uterine leiomyosarcoma was made. Re-staging CT brain, thorax, abdomen and pelvis and MRI brain were negative for local recurrence or distant metastases. She is currently undergoing radiotherapy to the scalp and surrounding tissues and will be followed up closely by the involved teams.

To the best of our knowledge, this is the first case described in the worldwide literature of isolated cutaneous metastasis to the scalp of uterine leiomyosarcoma without evidence of disseminated disease at other sites.

**Virtual slides:**

The virtual slide(s) for this article can be found here: http://www.diagnosticpathology.diagnomx.eu/vs/1311834987345566.

## Case History

**Figure 1:**
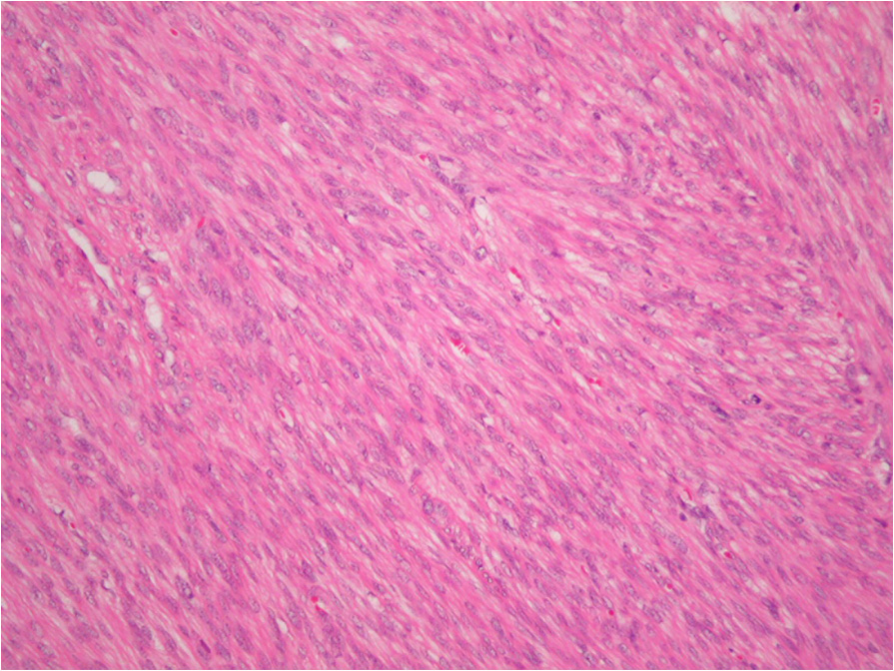
**Original uterine tumor specimen, H&E Section.** Spindle cell tumor with nuclear pleomorphism of abundant mitoses.

A 54 year old lady was referred to the surgical day ward of our institution for routine excision of a scalp lesion thought clinically to represent a sebaceous cyst of the right occiput. She had presented 4 years earlier with menorrhagia, fatigue and iron-deficiency anaemia and was diagnosed with a 9 cm fibroid uterus on ultrasound examination after inconclusive diagnostic laparoscopy. She underwent total abdominal hysterectomy and right salpingo-oophorectomy for 3 large uterine fibroids. Histo-pathological examination of the hysterectomy specimen revealed an incidental low-grade leiomyosarcoma (Figure 1). Staging CT thorax, abdomen and pelvis was negative for metastatic disease. She made an uneventful recovery and was treated further by adjuvant pelvic radiotherapy. Clinical and radiological follow up continued under the gynaecology and radiation oncology services and no objective evidence of metastatic disease was noted prior to the current presentation.

**Figure 2:**
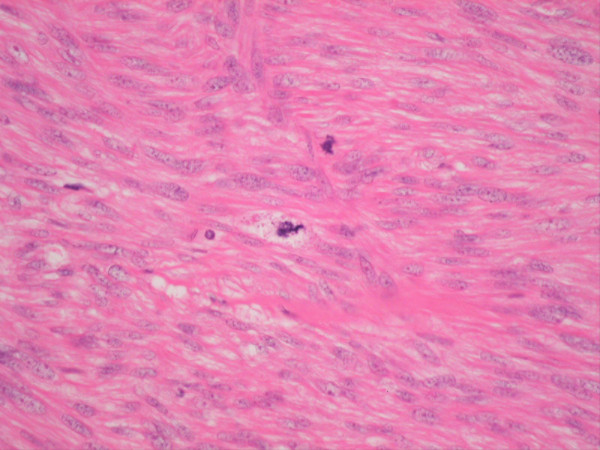
H&E Section shows cellular smooth muscle tumor with nuclear pleomorphism.

**Figure 3:**
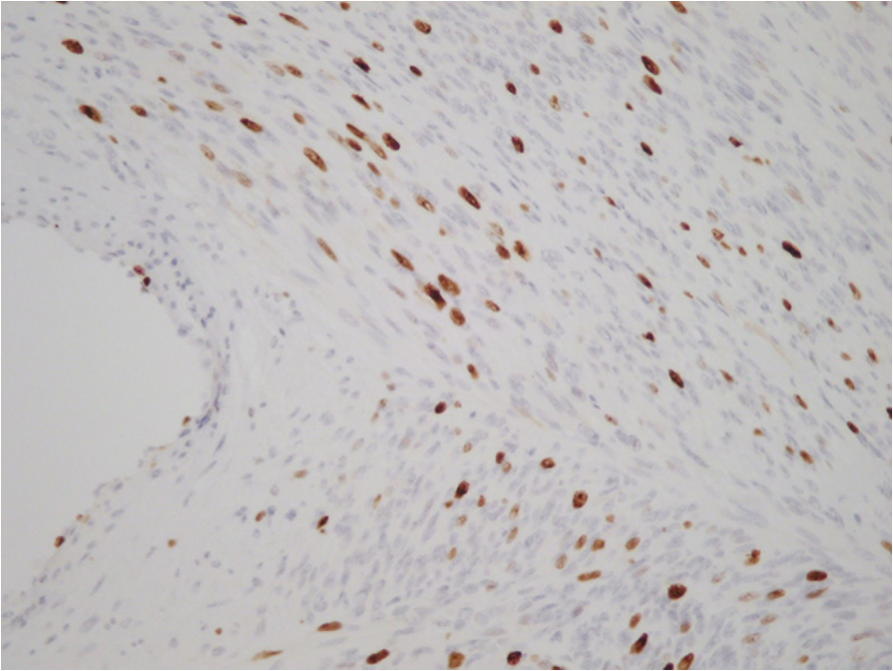
**H&E Section.** Atypical Mitoses.

**Figure 4:**
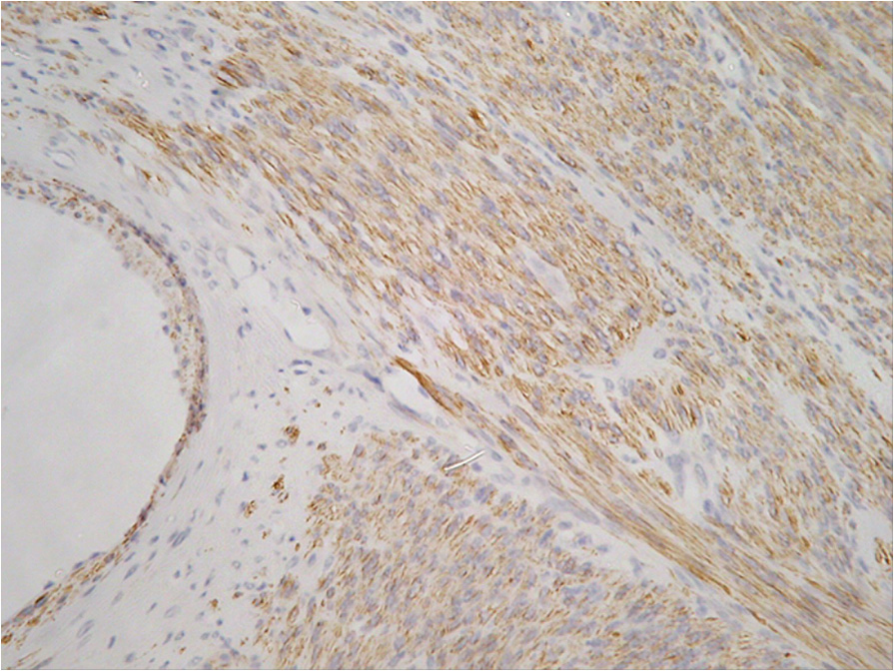
**Ki67.** High Proliferative Index.

**Figure 5:**
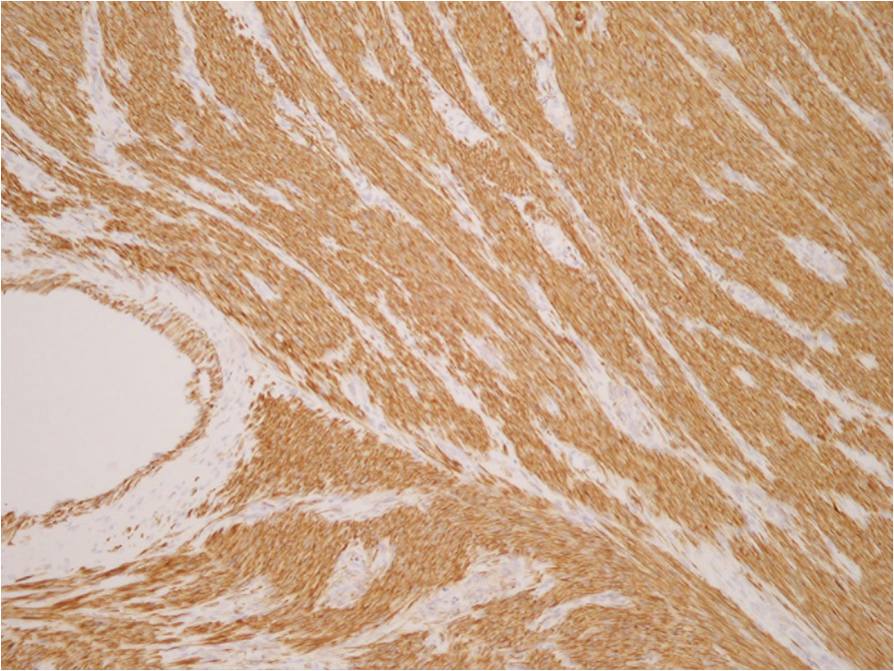
Actin.

**Figure 6:**
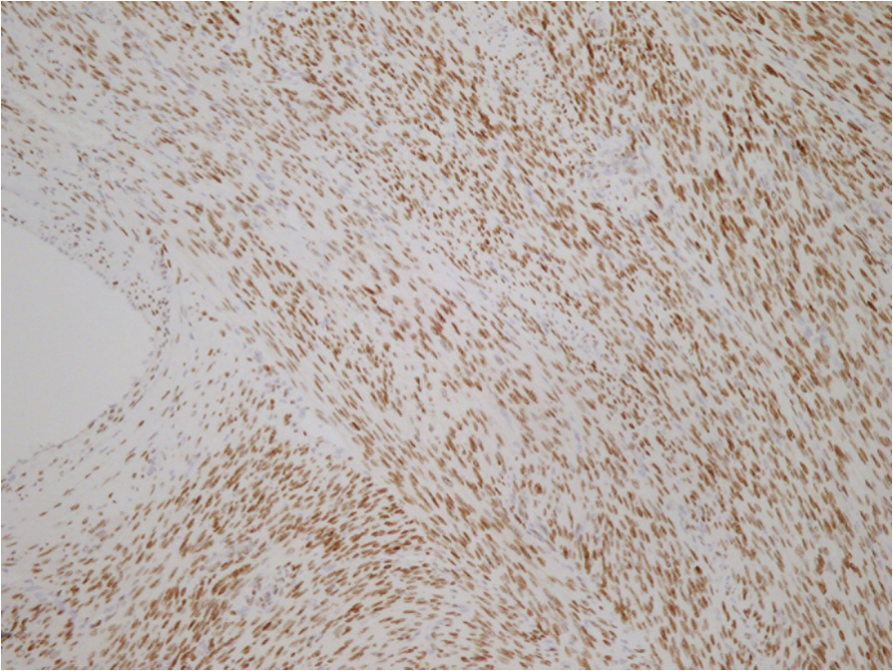
Desmin.

**Figure 7:**
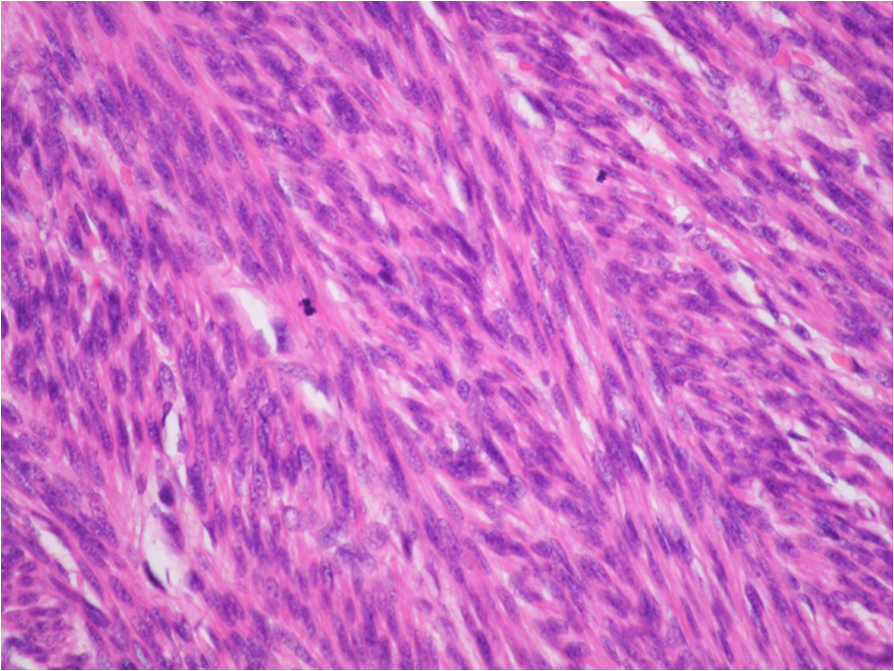
Oestrogen & Progesterone Receptor Positive.

She noticed an uncomfortable and unsightly cystic swelling on her occiput four years after hysterectomy and was referred for routine excision of what was believed to be a benign lesion. Following injection of local anaesthetic, the lesion was excised and the wound uneventfully closed with absorbable sutures. According to routine practice, the lesion was sent for histopathological examination. The wound healed well. Microscopic analysis including immuno-histochemistry demonstrated an ER and PR positive metastatic deposit of leiomyosarcoma (Figures 2, 3, 4, 5, 6 and 7). The margins of excision were histologically clear of disease.

At Multi-Disciplinary Team (MDT) discussion with input from pathologists, medical oncologists, radiation oncologists and surgeons, including review of the original hysterectomy sides, a confirmed diagnosis of metastatic scalp deposit from previous uterine leiomyosarcoma was made. Re-staging CT brain, thorax, abdomen and pelvis and MRI brain were negative for local recurrence or distant metastases. She is currently undergoing radiotherapy to the scalp and surrounding tissues, and may require salvage adjuvant chemotherapy at a future date if other sites of metastatic disease are detected.

## Discussion

Leiomyosarcoma is a rare and unpredictable malignancy of smooth muscle. Soft tissue sarcomas account for 0.7% of all malignancies and leiomyosarcoma accounts for 5-10% of these cases [1]. Leiomyosarcoma can occur in soft tissue, skin, blood vessels and bone [1], and are histologically similar irrespective of location. The most common site of leiomyosarcoma of soft tissue is the retroperitoneum, accounting for 50% of all cases [2]. Metastasis of sarcoma occurs by haematogenous spread [3], so cutaneous metastasis is an unusual discovery.

The definition of a cutaneous metastasis is “a neoplastic lesion arising from another neoplasm with which there is no longer continuity” [4]. These lesions account for 0.7% to 9% of all metastases [5]. More than half of cutaneous metastases arise from breast (51%) and almost one fifth (18%) from melanoma [6]. Cutaneous metastasis occurs less commonly in malignancies such as leukaemia and lymphoma, and metastasis of various types of sarcomas accounts for only 2–3% of all metastatic skin lesions [7].

Port site metastasis has been described in colorectal [8], gallbladder [9], gastric [10], ovarian [11] and lung cancer [12]. These tumors can be broadly categorised as cutaneous metastases but are entirely different in etiology and can be explained by transcoelomic spread or direct contamination [13,14].

Uterine leiomyosarcoma (ULMS) is an uncommon malignancy accounting for approximately 1% of uterine cancer [15] with an estimated annual incidence of 0.64 per 100,000 women [16]. Although leiomyosarcoma has also been described elsewhere in the pelvis including the cervix [17] and urinary bladder [18], it is more commonly found in the uterus. ULMS have high metastatic potential and 5 year survival rates vary from 0% to 73% in the literature [19-21]. Distant metastatic spread of ULMS has been described in lung [22], abdomen [23], soft tissue, and brain [24]. Less common metastatic sites include the breast [25] and bone [26]. Interestingly, another pathological entity, Benign Metastasizing Leiomyoma (BML) of the uterus describes metastasis of benign leiomyoma to the lung years post hysterectomy for uterine leiomyoma in pre-menopausal women. One study by Kayser et al [27]. examined 10 cases of BML and showed median survival was 94 months in BML patients and just 22 months in corresponding cases of confirmed lung metastasis of ULMS. BML patients can expect a much better prognosis than those with metastatic ULMS.

Cutaneous metastasis of ULMS is exceptionally rare and there is a paucity of information in the literature.

To the best of our knowledge only 4 cases of scalp metastasis of ULMS have been described worldwide [28-31]. The first reported case of ULMS with scalp metastases was described by Gardiner in 1917, and in this case the patient had perished and autopsy showed widespread metastasis involving the lungs, liver, pancreas and bone [28]. Another case involved disseminated intra-abdominal metastases and multiple cutaneous lesions [29]. A further case was described with cutaneous metastases involving both scalp and back [30]. In 2010 a case of primary ULMS with histologically confirmed scalp metastasis was published [31], but in this case there was also a history of disseminated disease from the primary uterine tumor and the patient had undergone pulmonary lobectomy for metastatic lung nodules. Our case of single cutaneous scalp metastasis from primary uterine leiomyosarcoma without evidence of other sites of disease spread is the first of its kind ever described worldwide.

One theory postulated by several authors explaining how a uterine leiomyosarcoma could metastasize to the scalp is haematogenous spread via Batson’s Plexus. This plexus was first described by anatomist Oscar Vivian Batson in 1940 and describes spread of tumour cells through the deep pelvic veins to the internal vertebral venous plexuses. Batson’s vertebral venous plexus consists of four interconnected venous networks surrounding the vertebral column. These valveless, thin walled, low pressure veins bypass the pulmonary, caval and portal venous systems, so this could provide an aberrant pathway of haematogenous spread by tumor in the pelvis to the head and neck [32-34].

The diagnostic value of progesterone receptor (PR) and p53 expression in ULMS is still under investigation. PR expression is useful in classifying leiomyosarcoma (LMS) vs. smooth muscle tumors of uncertain malignant potential (STUMP), Leiomyoma (LM) and Atypical Leiomyoma (ALM). A recent study [35] published in 2012 showed a prominent difference in PR expression between LMS and STUMP, and also demonstrated that ALMs had a similar PR staining pattern to LM. Thus, progesterone receptor expression can aid in effectively distinguishing both ALMs and STUMP from LMS. This study also showed low immunostaining rates of PR in LMS, which is consistent with previous studies. The study also examined the expression of p53, a tumor suppressor gene in LMS. Intense staining was described in the leiomyosarcoma samples, while other smooth muscle tumors, including STUMP, showed poor staining. Therefore p53 was shown clearly to be an indicator of malignancy. Overall PR and p53 staining allow for more accurate pathological diagnosis and therefore optimal management of such tumors can be achieved.

## Evidence basis for treatment

### (i) Surgery

Regarding treatment of recurrent or metastatic uterine leiomyosarcoma, surgical resection should be strongly considered in patients with localized single foci recurrences, either local or metastatic. In one study of 41 women who underwent resection for recurrent ULMS (17 local, 18 distant, 6 both), a two-year survival of 71% was reported in those patients who had a disease-free interval of 12 months or longer between resection of the primary tumor and the diagnosis of metastatic disease. Optimal surgical resection may provide an opportunity for long-term survival in these patients [36].

### (ii) Chemotherapy

Single agent doxorubicin has been shown to be somewhat successful in combating advanced ULMS. There have been two randomized trails showing the efficacy of doxorubicin single agent chemotherapy vs. combination chemotherapy. Doxorubicin proved as effective as a single agent vs. doxorubicin plus cyclphosphamide as response rates, and median overall survival (median 11.6 vs. 10.9 months) were similar in both arms for patients with measurable disease [37]. In another study comparing single agent Doxorubicin vs. combination Doxorubicin and Dacarbazine, Doxorubicin single agent therapy was also shown to as effective in terms of progression-free survival or overall survival (7.7 versus 7.3 months), although overall response rate was higher in the combination therapy patient group [38]. The most successful chemotherapeutic regime for combating advanced ULMS to date in patients with un-resectable uterine or other primary site ULMS is Gemcitabine and Docetaxel which has been shown to have an overall response rate of 53% and a mild toxicity profile, although this regime should be used in combination with a granulocyte colony stimulating factor to counteract neutropaenia [39].

### (iii) Radiotherapy

Postoperative adjuvant radiotherapy (RT) for advanced uterine leiomyosarcoma has been suggested by the European Organization for Research and Treatment of Cancer to have lower rates of local recurrence but no improvement in long term survival [40]. A retrospective study published in 1999, the largest series of its type, evaluated 103 women with uterine sarcoma (42% ULMS) who were treated with adjuvant radiotherapy. It was shown that irradiated patients had a higher rate of pelvic control than the control group (73% vs. 36%) combined with an increase in overall survival (73% vs. 37%) [41]. As yet there are no large studies evaluating the efficacy of radiotherapy in ULMS with confirmed distant metastases so each case must be discussed at multi-disciplinary team conference and managed accordingly.

Clinically, patients with cutaneous metastasis of leiomyosarcoma present with cyst like structures underneath the skin as in our case, or as cutaneous nodules [42]. In the context of a previous histological diagnosis of leiomyosarcoma caution should be exercised to remove any suspicious skin lesions for histopathological analysis. Due to the rarity of these cutaneous metastases and the lack of studies in this area, there is no specific guideline as to the optimal extent of an oncologically safe margin. Initial aggressive resection with wide margins is advised and restaging imaging is essential to rule out other metastatic disease. Although exceptionally rare, physicians, dermatologists and surgeons should keep in mind the metastatic potential of leiomyosarcoma to the skin.

## Consent

Written informed consent was obtained from the patient for publication of this Case Report and accompanying images.

## Competing interests

The authors declare there were no competing interests and the project was not funded.

## Authors’ contributions

SC drafted the manuscript. SC, AMH, WK and KB were involved in the surgical management of this patient. KB was responsible for conception and design of the case report. TN and FB made the histo-pathological diagnosis and provided images and slides for publication. FJS provided information related to the patient’s radiotherapy, follow up, and contributed valuable intellectual content. All authors read and approved the final manuscript.
